# Deep Learning and its Application for Healthcare Delivery in Low and Middle Income Countries

**DOI:** 10.3389/frai.2021.553987

**Published:** 2021-04-29

**Authors:** Douglas Williams, Heiko Hornung, Adi Nadimpalli, Ashton Peery

**Affiliations:** ^1^Harvard, MA, United States; ^2^D-tree International, Zanzibar, Tanzania; ^3^Baylor College of Medicine, Houston, TX, United States; ^4^D-tree International, Lincoln, MA, United States

**Keywords:** global health, NGOs, point of care diagnosis, digital health, machine learning, deep learning, artificial intelligence

## Abstract

As anyone who has witnessed firsthand knows, healthcare delivery in low-resource settings is fundamentally different from more affluent settings. Artificial Intelligence, including Machine Learning and more specifically Deep Learning, has made amazing advances over the past decade. Significant resources are now dedicated to problems in the field of medicine, but with the potential to further the digital divide by neglecting underserved areas and their specific context. In the general case, Deep Learning remains a complex technology requiring deep technical expertise. This paper explores advances within the narrower field of deep learning image analysis that reduces barriers to adoption and allows individuals with less specialized software skills to effectively employ these techniques. This enables a next wave of innovation, driven largely by problem domain expertise and the creative application of this technology to unaddressed concerns in LMIC settings. The paper also explores the central role of NGOs in problem identification, data acquisition and curation, and integration of new technologies into healthcare systems.

## Introduction

Over the past few years, most of the authors had the opportunity to work closely with the late Dr. Marc Mitchell (Marcus, 2019). Marc was one of the pioneers in digital health, recognizing how smartphones (and earlier PDAs), when combined with decision support protocols, can empower community healthcare workers, often with limited training, to effectively treat conditions such as pediatric pneumonia. As his work advanced, Dr. Mitchell helped define new ways evolving digital technologies can be deployed to strengthen healthcare systems.

As Dr. Mitchell advised, and as we later learned firsthand, healthcare delivery in Low and Middle Income Countries is fundamentally different from high-income economies. Patients may face challenging medical issues due to a complex interaction of lack of healthcare access, nutrition insecurity and economic instability, exhibiting conditions rarely seen in more affluent settings. Specifically, many common medical diagnostics and treatments are cost-prohibitive in LMIC settings, and severe skills and staffing shortages exist at all levels of care. For example, in 2011, it was reported that Malawi had roughly five OBGYNs providing care to a population of 14 million (Thorp, 2011). While that number has grown, and the University of Malawi College of Medicine now has an OBGYN residency program, an extreme skill shortage remains by developed world standards. Similar skills shortages exist in Uganda, where an estimated 20 dermatologists serve a population of 44 million (Health Volunteers Overseas, 2018).

While the strategic importance of AI in high-income economies is about improved quality of care and possibly lower costs, the strategic importance of AI in LMICs is about addressing critical medical skills and staff shortages, providing access to specialized skills, and empowering nurses and community healthcare workers (CHWs) to deliver services previously requiring scarce medical officers.

## Technical Background

### Artificial Intelligence, Machine Learning, and Deep Learning

For the non-technologist, the terms Artificial Intelligence (AI), Machine Learning (ML), and Deep Learning (DL) have been widely used, sometimes interchangeably, often leading to confusion.

**Artificial Intelligence** is the umbrella term for a broad, longstanding (Wikimedia Foundation, 2020) and aspirational field of research within Computer Science dealing with problems related to machine intelligence, such as mimicking cognitive functions, sensing environment, and taking independent action. Current fields of study include Robotics, Vision, Natural Languages, Learning, Planning, Reasoning, as well as others.

**Machine Learning** is a subfield of AI at the intersection of statistics and data mining, where a decision model is learned, rather than having been explicitly coded by a human. For example, traditional tools for helping Community Health Workers (CHWs) diagnose pediatric pneumonia may include hand-coded rule sets based on age and respiration rate. In a hypothetical tool using machine learning, it may be possible to derive more sophisticated decision trees based on a more complex and expanded set of features including weight, demographics and other vitals.

Traditional techniques (e.g., decision trees, SVM) can handle problems with hundreds or thousands of features. However, extensive preprocessing work is often needed to prepare the input data for the model, and for certain problems (e.g., image classification), these techniques can be insufficiently powerful.

**Deep Learning** (a.k.a. Deep Neural Networks) is a branch of Machine Learning where the mathematical models are inspired by the biological brain and excel at pattern recognition. Deep Learning has been successfully applied to problems such as Vision, Natural Language, Speech Recognition, Time series (e.g., ECG), Tabular, and Collaborative Filtering.

**Figure 1:**
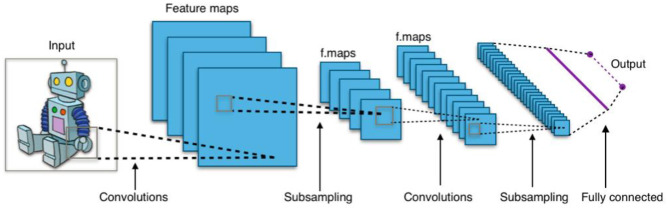
Typical 2D convolutional neural network. Image Source: By Aphex34 - Own work, CC BY-SA 4.0, https://commons.wikimedia.org/w/index.php?curid=45679374.

Without diving into much detail, these networks are composed of primitives call neurons, where a neuron is essentially a set of input values scaled by a set of “learned” weights, with an output based on some non-linear sum of these weighted values. Networks are typically organized as a succession of layers of neurons, with each layer providing input to the next. Figure 1 shows a simplified example of a convolutional neural network, a type of neural network commonly used for image processing applications (Waldrop, 2019). For example, lower-level maps might contain simple image features (e.g., corners, angled lines, color gradients), whereas higher-level maps detect increasingly complex features (e.g., branch-like objects, cloudy region) and interesting combinations of these features.

The RESNET-34 (He, 2015) image analysis model, for example, consists of 34 layers and >60,000 learned weight parameters. It is this large scale that makes these Deep Neural Models highly expressive.

For the lay person, an important aspect of deep learning is that the model extracts its own features automatically, layer by layer. This helps reduce or eliminate the complex preprocessing task (known as feature engineering) and leads to a better separation of concerns between those individuals focused on the problem domain (problem identification, gathering and labeling of data, and evaluation of overall suitability to task) and the technologists involved in the actual training of the model. Traditional drawbacks of deep learning are that development of bespoke models can require a high degree of technical sophistication, and training of these models can require large qualities of sample data.

**Transfer Learning** (Bengio, 2012; Yosinski, 2014; Tan, 2018) is based on a simple yet powerful observation that, for certain classes of deep learning problems, a large portion of a complex model is associated to feature extraction and that these extracted features are sufficiently generic to be useful beyond the direct problem at hand. This ability to build on the work of others radically reduces the technical complexity of developing a deep learning model and, from a strategic perspective, defines a particular technology sweet spot for NGOs considering the application of deep learning technology.

Transfer Learning is most effective for problems related to still 2D Images and Natural Language, where there is a significant degree of feature commonality across problems. In addition to still images, it is sometimes possible to transform other data into synthetic images and then employ transfer learning to the synthetic image. Potential examples of synthetic images include (1) transforming audio into spectrograms images (e.g., voice or cough), (2) creating cross-section images of video sequences [e.g., for detection of pleural sliding in lung point-of-care ultrasound devices (POCUS)], and (3) transforming one-dimensional ECG data into simple plot images of beat sequences.

### Illustrative Example: Pneumonia Detection

**Figure 2:**
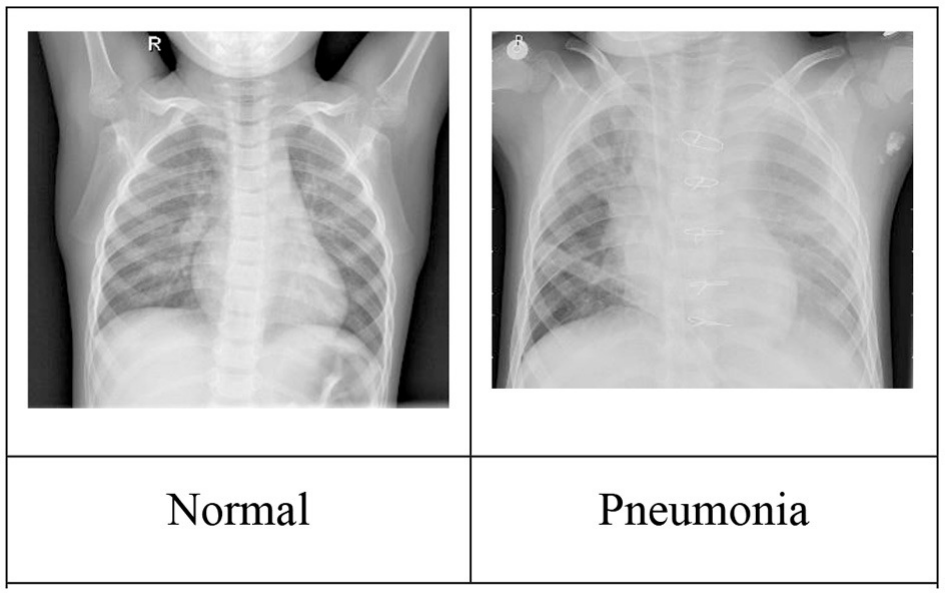
Sample chest X-ray images. Image source: Kermany (2018).

The Chest X-Ray Image Dataset^1^ (Kermany, 2018) contains 5,863 X-ray images (anterior–posterior) of 1–5-year-old pediatric patients categorized as Pneumonia/Normal, such as the sample images shown in Figure 2.

Table 1 shows the results of using Transfer Learning with a relatively small RESNET-18 model for various training set sizes. The model was pretrained, meaning the convolutional layer weights reuse values previously learned when RESNET18 was trained using the standard ImageNet Database (Deng, 2009). These pretrained values are provided as part of the fast.ai deep learning framework (Howard, 2020). While prediction quality generally improves with the number of training images^2^, it is notable that classifiers trained with relatively small numbers of images still deliver promising results.

**Table 1:** Classifier performance vs. training set size.

**Training set size (in images)**	**Sensitivity**	**Specificity**	**Accuracy**
1,000 Pneumonia/1,000 Normal	0.89	0.96	0.92
300 Pneumonia/300 Normal	0.97	0.81	0.89
200 Pneumonia/200 Normal	0.90	0.86	0.88
100 Pneumonia/100 Normal	0.90	0.88	0.89
50 Pneumonia/50 Normal	0.98	0.80	0.89

For a relatively small global health NGO considering AI, the above example shows the potential of starting small, delivering an initial good-enough solution, then iteratively improving the solution over time with additional data. Of course, “good-enough” is a medical decision, not a technical one, and data requirements for achieving an initial minimally viable solution will vary by application space.

While the above results are for illustrative purposes and are not a state of the art (Kaggle, 2019), they are indicative of what may be achievable by non-specialists with a modest level of effort, and no prior background in this medical domain.

As the technical aspects of producing high-quality prediction models become increasingly turnkey, it is the availability of relevant diagnostic image datasets that is the strategic enabler. Medical domain expertise is essential to the gathering of these datasets, providing ground-truth assessments, and evaluating accuracy of predicted results. These datasets will need to be actively curated, augmenting the dataset to address prediction blind spots, such as situations where there are insufficient examples for proper training, or in ensuring adequate diversity of examples when patient demographics are an issue.

Much of the potential training data that are widely available, such as the example above, naturally skew toward use-cases most relevant to higher resource settings. While X-ray imaging is ubiquitous in high-resource settings, basic x-ray capability may not be present in many district level hospitals in LMIC settings. Smartphone connected low-cost POCUS can be an attractive alternative for pneumonia diagnosis (MSF, 2018). Under these situations, the machine learning classifier would be trained to detect A-Lines, B-Lines, and Pleural Sliding as components of the overall diagnosis process (Nadimpalli, 2019). Similarly, CT is emerging as the technology-of-choice for monitoring the progression of COVID-19 in high-resource settings, whereas tools to assist in COVID-19 assessment using POCUS (Buonsenso, 2020; Soldati, 2020) may be more relevant in LMIC.

## Global Health Implications

### Ministries of Health (MoHs) and Non-Governmental Organizations (NGOs)

MoHs, supported by NGOs, have historically taken the lead in providing and improving healthcare for populations. In low-resource settings, there are multiple gaps—from qualified Human Resources (as described above in Malawi and Uganda) to availability of field-relevant diagnostics. One solution to this has been the concept of task-shifting, where non-specialists perform the tasks of specialists through protocolized medicine. Examples of task-shifting can be found in medical fields as diverse as surgery (Falk, 2020) to dermatology (Brown, 2018). Artificial Intelligence-based tools can provide improved support to ensure that task-shifting is safe and effective for patients. Moreover, because many diseases are unique to low-resource settings where disease profiles and prevalence are unique (Kaplan, 2010), targeted AI applications based on locally sourced data can potentially improve the individualization of protocols to each patient. However, before AI can be deployed in these settings, MoHs and NGOs need to consider the potential uses, the risks and also the stakeholders who may be affected.

As described above, AI and its subset of Transfer Learning can help with the diagnosis of pneumonias based on chest x-rays in a relatively straightforward manner. Other successful applications of AI can be seen in helping diagnose tuberculosis in X-rays (Qin, 2019) and finding signs of severe malaria on retinal images (Joshi, 2012). Many more ideas can be considered—based solely on the needs and imagination of clinicians and programmers worldwide. Broadly speaking, there are three key areas in which AI can assist in global health: (a) clinical decision support at both health center and community levels, (b) population health, and (c) direct patient support (USAID, 2019).

### Medical Technology Companies and Universities

Universities and (medical) technology companies are the institutions that produce new theories, methods, and techniques that can result in new solutions for healthcare delivery challenges. This capacity comes with opportunities and responsibilities. We have written about opportunities in the first part of this paper. The responsibility of these institutions is to propose solutions that solve local problems without introducing unintended negative side effects. Global Health does not mean that solutions developed in a lab setting or within a specific socio-economic or cultural context work in different local settings. For example, algorithms for diagnosing cancerous skin lesions need to be sensitive to variations in presentations by skin color (Bradford, 2009; Kundu, 2013), and the application of a method or technique that works well in the lab or in a high-income-country setting (Phillips, 2019) may require additional validation and potentially supplemental training when used with substantially different patient populations. Additionally, tools can fail due to differences in clinical setting such as background noise or ambient light, or technology that is too sensitive to user training and errors (e.g., Beede et al., 2020).

Applying “solutions” without considering the local context can result in low acceptability or even introduce risks and harm to people receiving care. Furthermore, besides the immediate application of a solution, the solution's long-term sustainability and integration into an existing (digital) health system needs to be considered to not cause negative effects. A technological solution that does not consider health worker workflows or the reality of patients might result in low acceptability, e.g., if the local workflows require extra steps, if the technology makes the healthcare worker look less competent, or if the technology requires the patient to move to facilities where they experience a high travel burden, long waiting times, etc. A predictive algorithm for risk assessment might make a healthcare worker focus on true and false positives and neglect true and false negatives, which will result in negative individual and possibly in negative public health outcomes.

To adequately consider the local context, a research or technology institution needs to be rooted locally or have a strong collaboration with local institutions. If a problem is not trivial or truly universal, the assumption should be that a “solution” not developed for a specific local context will not work without adjustments, because the local problem is substantially different from the original problem. Besides local academic or industry rootedness or collaboration, locally operating digital health NGOs or development partners can play important roles: they understand the local context and can thus identify which AI technologies and investments will be effective; they can contribute to making data available in the right format, adequately considering consent of the “data subjects,” and guaranteeing that MoHs as public health data owners understand and are part of the data value chain; they understand and can propose incremental solutions where AI or machine learning is introduced gradually (e.g., in telemedicine systems where data are already captured and algorithmic solutions can increasingly augment human diagnoses); they understand that AI-enabled solutions require local skills and processes to guarantee sustainability in terms of, e.g., lifecycle management and integration into the local digital health system.

### Smartphones as Machine Learning Deployment Platform

One physical tool that has significant synergies with using AI in low-resource settings is the smartphone. For the past two decades, low-cost smartphones, and their PDA predecessors, have played an important role in LMIC healthcare delivery. In addition to integration with patient case management systems (Ollis, 2016), these devices have hosted important decision support tools for family planning (Agarwal, 2016), prenatal and antenatal care (Hackett, 2018), intrapartum progression of labor (Sanghvi, 2019), diagnosis of pediatric pneumonia (Mitchell, 2013), AIDS (Mitchell, 2009) treatment, and many others.

The combination of user interface, communications capabilities, audio and video sensors, and local computational capabilities (Ignatov, 2018) makes modern smartphones an attractive platform, providing input for next-generation machine learning-based applications. The included camera can be a powerful tool for image capture, either when used directly or when supplemented with an external device. For skin lesion analysis, add-on external lenses with polarized light source are available at relatively low cost^3^. Fundus images can be captured using the native camera along with indirect condenser lens (Tran, 2018), with potential applicability for diagnosis of diabetic retinopathy and cerebral malaria (Bear, 2006).

In addition to a smartphone's integrated camera, external cameras can be used to enable additional use-cases. Two types of low-cost (<$50) consumer devices of potential relevance are digital otoscope/endoscope devices (for eye/ear/nose/mouth exam) and digital microscopes (for lab specimen analysis). While these devices may have much lower resolution than the native smartphone camera, the device resolution may still be good-enough for many tasks. Other peripherals, such as low-cost point-of-care ultrasound discussed earlier, can significantly extend the phone's native capabilities.

### General Advice on Implementing AI for MoHs and NGOs

In implementing AI in low-resource settings, it would be best to use iterative, field-based processes that are built into existing systems and institutions rather than starting from scratch, or hoping to replace existing systems, however broken—an institution that cannot fix itself is unlikely to be able to support and use a complex technology properly (Weber, 2010). Moreover, there are inherent risks particular to AI. Four key ones are the following:

1. Ground truth—as described above, Supervised Learning relies on labeled data, which in turn relies on the both the quality of the underlying data and the appropriate expert labeling—both of which can be scarce in low-resource settings.
2. Generalizability—AI algorithms built on one dataset may not necessarily be applicable to other datasets (Kelly, 2019). The heterogeneity of populations in low-resource settings and the variable prevalences of health indices such as malnutrition and HIV can add complexity and decrease the transferability of algorithms between populations. This may necessitate a minimum portion of local data to be incorporated into model training.
3. Data ownership and confidentiality—there is currently an active debate about personal data ownership outside of the medical field, especially involving large corporations (McNamee, 2019). Similar arguments are starting to happen with AI and the medical field (Lomas, 2016). In pursuing AI for health-related issues, MoHs and NGOs should follow the highest ethical standards, ensuring full transparency with patients and anonymity where needed, creating clear data transfer and ownership policies, and enabling creative ways of ensuring that future algorithms are open and affordable to all users, such as through open-prize competitions^4^ and open-access database repositories^5^.
4. Localization—if not already developed for a specific local context, AI-enabled solutions need to be evaluated and adapted to the local socio-environmental, technological, and organizational reality. This process requires collaboration with local government, academic, and potentially industry partners. Locally active digital health NGOs can play a role as facilitator or driver of this collaboration.

Fundamentally, there are significant benefits to using AI to help patients and populations in low-resource settings. AI is feasible and can be a powerful tool. However, every organization should map the key stakeholders, including patients, communities, policy makers, and healthcare providers, to ensure that before deployment of AI, there are clear expectations and objectives. These objectives should be realistic and iterative within the context of existing structures and should mitigate potential risks to patients through a transparent process.

## Conclusions

NGOs are uniquely positioned to help ensure that LMICs benefit in the era of AI and are not left behind. While non-trivial, AI technologies such as image classification have sufficiently matured that it is now possible for even smaller NGOs to engage AI projects without developing deep in-house technical experience. As was learned during the earlier generation of smartphone-based healthcare tools, it is critical to consider local needs, demographics, customs, workforce characteristics, existing/planned health system components, and conditions on the ground. It is this simultaneous ability to know what problems to solve, and how best to ultimate deploy these solutions, where NGOs should add significant value. Otherwise, the field will repeat mistakes made during previous technological innovation iterations that led to fragmented and unsustainable components that did not integrate into existing systems. As discussed earlier, AI technology has strong ethical considerations, and it is critical that NGOs be mindful of these concerns, putting in place the proper safeguards to ensure that this powerful technology is harnessed for good.

## Author Contributions

This paper is the product of a collaboration between DW and HH (technology) and AN (medicine) with AP providing valuable feedback and edits. All authors contributed to the article and approved the submitted version.

## Conflict of Interest

The authors declare that the research was conducted in the absence of any commercial or financial relationships that could be construed as a potential conflict of interest.

## Footnotes

^1^https://www.kaggle.com/paultimothymooney/chest-xray-pneumonia.

^2^In certain instances, results improved at smaller training set size. This is most likely due to noise related to variability in composition of these smaller datasets.

^3^https://dermlite.com/products/dermlite-hud.

^4^https://stanfordmlgroup.github.io/.

^5^https://www.kaggle.com/datasets (accessed April 13, 2021).
